# *In vitro* adherence of *Staphylococcus pseudintermedius* to canine corneocytes is influenced by colonization status of corneocyte donors

**DOI:** 10.1186/1297-9716-44-52

**Published:** 2013-07-08

**Authors:** Narayan Chandra Paul, Francesca Latronico, Arshnee Moodley, Søren Saxmose Nielsen, Peter Damborg, Luca Guardabassi

**Affiliations:** 1 Department of Veterinary Disease Biology, Faculty of Health and Medical Sciences, University of Copenhagen, Stigbøjlen 4, 1870 Frederiksberg C, Denmark; 2 Department of Large Animal Science, Faculty of Health and Medical Sciences, University of Copenhagen, Groennegaardsvej 8, 1870 Frederiksberg C, Denmark

## Abstract

The current knowledge of *in vitro* adherence of *Staphylococcus pseudintermedius* to canine corneocytes is limited to comparative analyses between strains, staphylococcal species or corneocytes collected from different breeds, body sites and hosts. However, the role played by colonization status of corneocyte donors remains unknown. The aim of this study was to evaluate the adherence properties of commensal *S. pseudintermedius* strains to corneocytes collected from dogs with different colonization status. For this purpose, corneocytes were collected from five dogs that were classified as persistently colonized (D1 and D2), intermittently colonized (D3 and D4) or non-colonized (D5) on the basis of the results of a previous longitudinal study. Adherence to corneocytes originating from each of the five dogs was assessed by an *in vitro* adhesion assay using four genetically unrelated strains isolated from the colonized dogs (S1 to S4). Irrespective of their host of origin, all strains adhered significantly better to corneocytes from D1 and D2 than to corneocytes from D3, D4 and D5 (*P* < 0.0001). The mean count of cells adhering to corneocytes from persistently colonized dogs was on average three times higher than the mean count using corneocytes from the other dogs. A significant difference between strains was only observed for one strain-corneocyte combination (S2-D4), indicating that *S. pseudintermedius* adherence to corneocytes is driven by host factors and only marginally influenced by strain factors. This finding has important implications for understanding and preventing *S. pseudintermedius* skin colonization and infection.

## Introduction

*Staphylococcus pseudintermedius* is an opportunistic pathogen that resides on the skin and mucosae of dogs and other members of the Canidae. The percentage of *S. pseudintermedius* carriage reported in the scientific literature ranges between 46 and 92% among healthy dogs [1]. We have previously shown that most dogs are either persistent or intermittent carriers, and very few individuals are non-carriers over a period of one year [2].

Staphylococcal adherence to corneocytes is a crucial step for skin colonization and subsequent infection. Previous studies of *S. pseudintermedius* adhesion to canine corneocytes mainly focused on differences between strains, staphylococcal species, dog breeds, body sites and disease status [3-5]. *S. pseudintermedius* adheres better to corneocytes from dogs suffering atopic dermatitis or belonging to specific breeds, and adherence to canine corneocytes by this species is greater than for other staphylococcal species typically associated with humans such as *S. aureus* and *S. hominis*[3,5]. Measurement of adherence is influenced by incubation conditions and bacterial concentrations used in the laboratory assay but no significant difference has been found between *S. pseudintermedius* isolates from pyoderma lesions and isolates from healthy dogs, suggesting a lack of correlation between virulence and *in vitro* adherence to canine corneocytes [4]. However, none of these studies considered the possible role played by the colonization status of the dogs from which corneocytes were collected. In order to fill this knowledge gap, the aim of this study was to evaluate the adherence properties of commensal *S. pseudintermedius* strains to corneocytes collected from dogs with different colonization status. *In vitro* adherence was assessed using strains and corneocytes collected from persistently colonized, intermittently colonized and non-colonized dogs from a previous longitudinal study.

## Materials and methods

### Selection of dogs

Five dogs from a previous longitudinal study [2] were included in this study: two persistent carriers (D1, D2), two intermittent carriers (D3 and D4) and one non-carrier (D5). The dogs were classified into different carriage groups on the basis of the analysis of nine samples collected over a period of six months. Dogs were defined as persistent carriers (positive for *S. pseudintermedius* at all sampling times), intermittent carriers (positive in at least one sample) and non-carriers (negative at all sampling times). The colonization status of the dogs was confirmed when corneocytes were collected, approximately nine months after the end of the longitudinal study; *S. pseudintermedius* was isolated from both the mouth and the perineum of D1, D2 and D3, only from the perineum of D4, and from none of the two body sites of D5. Dogs were defined as healthy by physical examination and had no history of antimicrobial therapy and skin infection in the last six months prior to collection of corneocytes. Data on breed, sex, age and colonization status of the five dogs are summarized in Table 1.

**Table 1:** Data of the five dogs from which strains and corneocytes were collected

**Dog ID**	**Breed**	**Age (months)**	**Sex**	***S. pseudintermedius *carriage status**	**Strain**	**MLST type**	**PFGE type**
D1	Labrador retriever	25	Male	Persistent carrier	S1	ST165	Distinct
D2	German shepherd	69	Neutered female	Persistent carrier	S2	ST138	Distinct
D3	Labrador retriever	39	Female	Intermittent carrier	S3	New ST	Distinct
D4	Border collie	28	Male	Intermittent carrier	S4	ST20	Distinct
D5	Saluki	64	Female	Non-carrier	-	NA	NA

*MLST* multi-locus sequence typing, *ST* sequence type, *PFGE* pulsed-field gel electrophoresis, *NA* not applicable.

### Collection of corneocytes

Corneocytes were collected by veterinary staff during August 2012, after obtaining informed consent from the owners. Corneocytes were collected from the inner pinna of the ear by applying adhesive discs. Before collection, surface debris was removed by applying five successive adhesive tape strips (Sellotape^®^ Original, Winsford, Cheshire, UK). Corneocytes were collected by pressing 25-mm diameter adhesive discs (Dsquame^®^, CuDerm Corporation, Dallas, TX, USA) to the cleaned area on both ears. The discs were then carefully removed to obtain confluent corneocyte layers and placed in sterile petri dishes. From each dog, 12–16 corneocyte samples were collected at the same time. Immediately after collection, all the corneocyte samples were transferred to the laboratory and stored at 4°C. All slides were examined by light microscopy, and only slides with confluent corneocytes were included in the experiment.

### Bacterial strains

The four *S. pseudintermedius* strains used in this study (S1, S2, S3 and S4) were previously isolated from dogs D1, D2, D3 and D4 [2]. All strains were genetically distinct by pulsed-field gel electrophoresis [6] and multi-locus sequence typing [7]. The genetic characteristics of the strains are described in Table 1.

### Corneocytes adherence assay

The four bacterial strains were tested with all five dogs’ corneocytes. The adhesion assay was done according to Moodley et al. [8] with minor modifications in the centrifugation condition (3000 rpm for 3 min) and the adjusted OD_600_ bacterial suspension ((OD)_600_ = 0.15 (~7 × 10^7^ CFU/mL)). Discs were examined manually using light microscopy to count adhered bacteria at × 1000 magnification on a Axioplan II epifluorescence microscope (Zeiss, Oberkochen, Germany) and a Zeiss AxioCam digital camera. Ten microscopic field images with good confluent corneocytes were selected and used to count the adhered bacteria. All experiments were done in duplicate except for dog D1 and strain S3 because there were not sufficient slides with confluent corneocytes. One corneocytes disc per individual was incubated with PBS only and included as a negative control. The adhesion assay for each strain was done at the same time and performed by the same person to avoid variations in counting adherent bacteria.

### Statistical analysis

Descriptive statistics were computed as the lower quartile, the median and the upper quartile for each bacterial strain adhered with each dog's corneocytes. Then, the mean adhered bacterial count of the persistent carrier dog corneocytes was compared to the mean count from non-persistent dogs with a negative binomial regression model using the Genmod procedure in SAS version 9.3 (SAS Institute, Cary, NC, USA). The model was also used to determine if there was any preferential binding of a particular strain to corneocytes from individual dogs, and if a particular strain adhered better to corneocytes in general.

## Results

**Figure 1:**
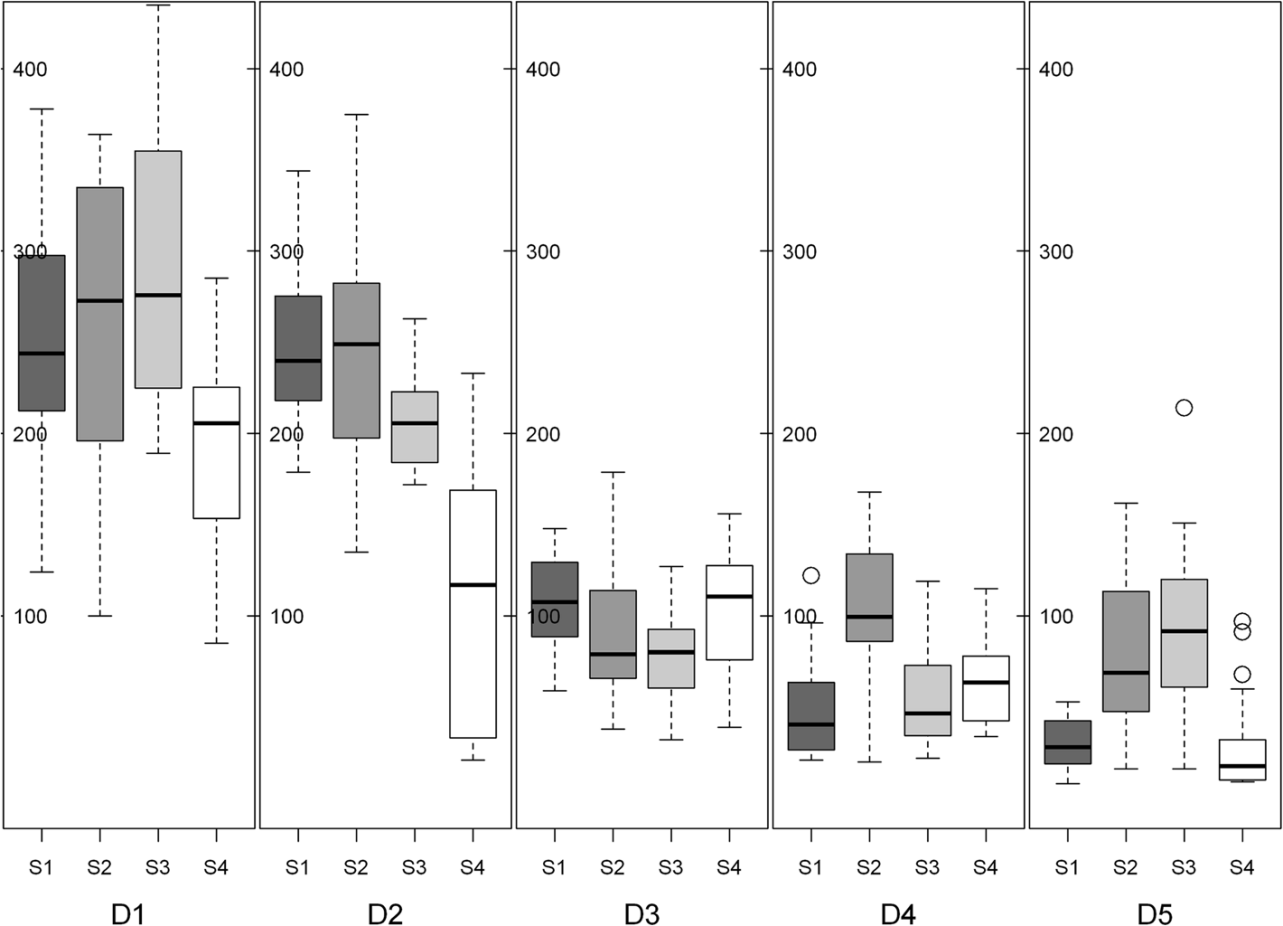
**Summary statistics of adherence of *S. pseudintermedius *to corneocytes.** Boxplots illustrating the count of four *Staphylococcus pseudintermedius* strains (S1-S4) adhering to corneocytes collected from five different dogs (D1-D5). The counts (y-axis) express the summary statistics of number of adhered bacteria per microscopic field for each dog-strain combination. The line within each box represents median; the bottom and top lines of the box represent lower quartile (Q1) and upper quartile (Q3). D1 and D2, persistent carriers; D3 and D4, intermittent carriers; D5, non-carrier; S1-S4, strains from dogs D1-D4.

**Figure 2:**
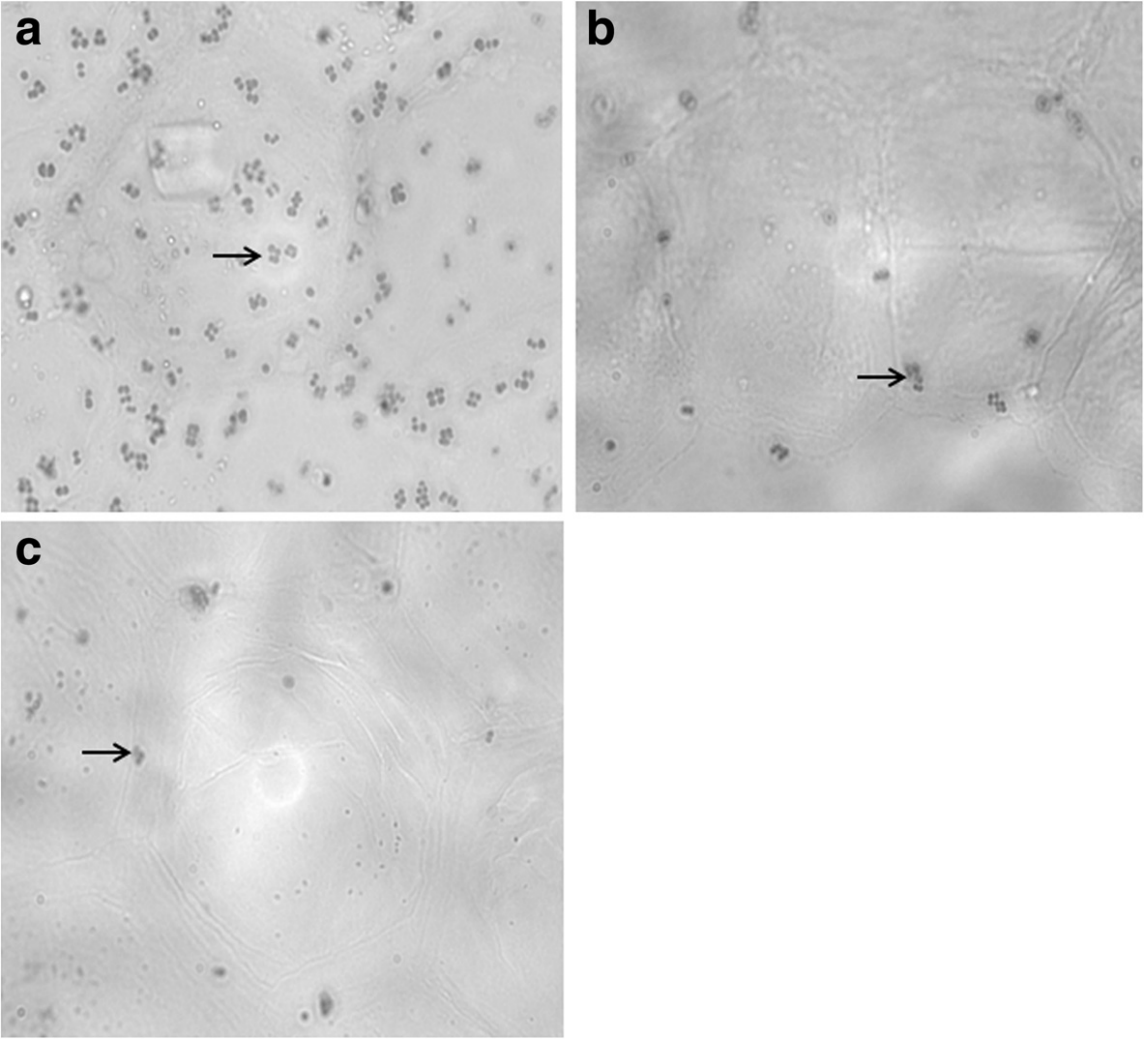
**Adherence of *Staphylococcus pseudintermedius *to corneocytes.** Microscopic image (× 1000 magnification) of *S. pseudintermedius* strain S2 adhering to corneocytes of: **a)** a persistent carrier (D1), **b)** an intermittent carrier (D3), and **c)** a non-carrier (D5). Arrow signs indicate examples of bacteria adhered to canine corneocytes.

Boxplots for adherence values of the four *S. pseudintermedius* strains to the corneocytes from the five dogs are illustrated in Figure 1. All *S. pseudintermedius* strains adhered significantly better to the corneocytes from both persistent carriers (D1 and D2) in comparison to those from the two intermittent carriers (D3 and D4) and the non-carrier (D5) in the negative binomial model (*P* < 0.0001). The mean count of bacteria adhering to corneocytes from D1 and D2 was 224 cells per microscope field (95% confidence interval: 193–258), whereas the mean count using corneocytes from non-persistent carriers was only 74 cells per field (95% confidence interval: 61–89). Three representative photographs of bacterial adhesion to corneocytes with different colonization status are shown in Figure 2.

A significant difference between strains was only observed for one strain-corneocyte combination. Strain S2 adhered better to corneocytes from D4 compared to the other three strains, including strain S4 isolated from this dog. No bacteria were observed in the negative control slides, confirming absence of bacteria on the collected corneocytes.

## Discussion

This study provides useful knowledge on the variability of *S. pseudintermedius* adherence to corneocytes from dogs with different colonization status. The results indicate a greater adhesion of *S. pseudintermedius* to corneocytes from persistent carriers than intermittent carriers or non-carriers, regardless if the strain was resident or non-resident in the dog from which the corneocytes were collected. To our knowledge, this is the first report showing that adherence of *S. pseudintermedius* to canine corneocytes is associated with colonization status of the corneocyte donor.

Staphylococcal adhesion to corneocytes is the first step of colonization and a complex biological process involving host and strain factors. A recent study by Moodley et al. [8] showed that *S. aureus* adhered better to corneocytes from colonized pigs than from non-carriers. Similarly, adherence of *S. aureus* to nasal mucosal cells from human *S. aureus* carriers was greater than to cells from non-carriers [9]. In humans, host factors such as polymorphisms in the glucocorticoid receptor gene [10], interleukin 4, complement factor H, C-reactive genes [11], and distinct histocompatibility antigens [12] have been shown to play an important role in *S. aureus* carriage. Similarly, canine host genetic factors may also play a role in *S. pseudintermedius* carriage. Greater adherence by *S. pseudintermedius* to corneocytes from atopic skin was observed when compared to corneocytes from healthy skin [5,13]. This may be due to the changes of skin receptors specific for staphylococci, greater expression of intercellular adhesion and different distribution pattern of fibronectin in atopic skin [13-15].

Breed is an additional host factor that has previously been shown to affect adherence of *S. pseudintermedius*, with greater adherence to corneocytes from breeds such as Boxer and Bull Terriers [3]. As we only studied five dogs belonging to four breeds, this study was not designed to evaluate the role played by this host factor. However, corneocytes from two dogs belonging to the same breed (Labrador Retriever, D1 and D3) displayed different adherence patterns correlating to the colonization status of the two dogs, therefore suggesting that colonization status may play a more important role than breed in *S. pseudintermedius* adherence to corneocytes.

Adhesins are virulence factors that determine the ability of bacteria to adhere to the host tissues [16]. Significant differences in adherence have been reported between strains of *S. pseudintermedius* with canine corneocytes [4] as well as between strains of *S. aureus* with porcine corneocytes [8]. However, Saijonmaa-Koulumies and Lloyd suggested there is no correlation between virulence and *in vitro* adherence patterns of *S. pseudintermedius*[4]. In our study, the strains were genetically distinct and no preferential binding was shown by one strain over the others, with the exception of a particular strain-corneocyte combination (S2-D4). Interestingly this exception did not regard strain and corneocytes from the same dog, showing that preferential binding is not influenced by strain adaptation to individual hosts. Thus, although based on a limited number of dogs, our findings provide a clear indication that *S. pseudintermedius* adherence to canine corneocytes is driven by host factors and only marginally influenced by strain factors. It remains to be determined whether corneocyte adhesion is an important factor for colonization or for initiation of clinical infection. It is not unlikely that mucosal surfaces are the primary colonization site for *S. pseudintermedius* and that skin is repeatedly contaminated by bacteria from mucosal sites. If this hypothesis is true, corneocyte adhesion would be indicative of host predisposition to skin infection rather than colonization. More research is needed to clarify the biological significance of corneocyte adhesion in relation to canine colonization and infection.

*S. aureus* colonization has been shown to be a risk factor for human infection [17]. Persistent carriers have higher *S. aureus* loads on the nasal mucosa and are more likely to become infected compared to non-carriers [18]. Similarly, persistently colonized dogs harbour higher numbers of *S. pseudintermedius* on the oral mucosa [19] and might have a greater risk for infection. The higher frequency of *S. pseudintermedius* colonization in atopic dogs in comparison to healthy dogs [20] suggests that staphylococcal colonization plays an important role in canine pyoderma. Future research is warranted to identify possible host genetic factors predisposing to *S. pseudintermedius* colonization. This information may be used for developing alternative strategies for prevention of staphylococcal infections in dogs. Dog breeds have been created or modified by man to select for specific phenotypic traits such as size, shape, coat colour and behavior. Thus, it is not unrealistic to hypothesize that breeding programmes could be designed to select for dogs with reduced susceptibility to *S. pseudintermedius* colonization.

## Competing interests

The authors declare that they have no competing interests.

## Authors’ contributions

NCP, AM, PD and LG: Conceived and designed the experiment. NCP and FL: Performed the experiment. NCP and SSN: Analysed the data. NCP, FL, AM, PD, SSN and LG: Wrote and approved the final manuscript. All authors read and approved the final manuscript.
